# Assessing the performance of paediatric early warning scores to predict critical deterioration events in hospitalised children (the DETECT study): a retrospective matched case-control study

**DOI:** 10.1186/s12887-025-05754-x

**Published:** 2025-07-02

**Authors:** Abbey Bracken, Steven Lane, Sarah Siner, Dawn Jones, Caroline Lambert, Fulya Mehta, Chin-Kien Eyton-Chong, Peter Davis, John Fitzsimons, Emma Lim, Linda Clerihew, Bernie Carter, Gerri Sefton, Enitan D. Carrol

**Affiliations:** 1 Department of Clinical Infection, Microbiology and Immunology, Institute of Infection, Veterinary and Ecological Sciences, University of Liverpool, Liverpool, UK; 2 Health Data Science, Institute of Population Health, University of Liverpool, Liverpool, UK; 3 Clinical Research Division, Alder Hey Children’s NHS Foundation Trust, Liverpool, UK; 4 Department of General Paediatrics, Alder Hey Children’s NHS Foundation Trust, Liverpool, UK; 5 Critical Care Unit, Alder Hey Children’s NHS Foundation Trust, Liverpool, UK; 6 Bristol Royal Hospital for Children, Bristol, UK; 7 Children’s Health Ireland at Temple Street, Dublin, Ireland; 8 Department of General Paediatrics, Great North Children’s Hospital, Newcastle upon Tyne, UK; 9 Population Health Sciences Institute, Newcastle University, Newcastle upon Tyne, UK; 10 Ninewells Hospital, Dundee, UK; 11 Faculty of Health, Social Care and Medicine, Edge Hill University, Ormskirk, UK; 12 Paediatric Intensive Care Unit, Alder Hey Children’s NHS Foundation Trust, Liverpool, UK

**Keywords:** PEWS, Paediatric early warning score, Critical deterioration events, Critical care, Admission

## Abstract

**Background:**

Paediatric Early Warning Scores (PEWS) enhance patient safety, by focused monitoring of vital signs to identify children at risk of deteriorating. However, there is an acknowledged need for standardisation. The aim of this study was to compare the performance of seven PEWS (Alder Hey, Bedside, Bristol, Irish, Newcastle, Scottish and the proposed National PEWS for England (v3)) utilised in clinical practice in the United Kingdom and Ireland. The primary outcome was occurrence of a critical deterioration event (CDE) in hospitalised children, and secondary outcome was 72-hour hospital mortality.

**Methods:**

250 patient episodes were identified over a 12-month period. Cases were matched 2:1 with controls; using age range and admitting specialty. PEWS were calculated, along with performance characteristics. Maximum PEWS were calculated at 24, 12, 6 and 4 h prior to CDE or discharge, and area under the receiver operating curve (AUC) used to measure performance. Sub-group analysis explored performance within 3 specialities observed to have increased risk for deterioration. Kaplan-Meier survival curves compared time to event data using the identified optimum PEWS performance cut-point.

**Results:**

The median age of patients experiencing CDE was 8 months. AUCs across all PEWS in predicting CDE, ranged from 0·87 to 0·95. Optimum cut-offs for each PEWS were identified. Kaplan-Meier curves for cumulative risk of time to CDE according to the PEWS stratification, demonstrated CDE was significantly less likely for patients below the cut-off values (log-rank test, *p* < 0·001).

**Conclusions:**

All seven PEWS assessed demonstrate excellent predictive ability for CDE, in a heterogenous cohort. For evaluation of PEWS performance, CDE is a more appropriate outcome measure than hospital mortality, due to low mortality outside PICU. A standardised PEWS allows consistency, benchmarking and opportunity for continuing recalibration.

**Supplementary Information:**

The online version contains supplementary material available at 10.1186/s12887-025-05754-x.

## Introduction

The UK Confidential Enquiry into Maternal and Child Health report ‘Why Children Die: A Pilot Study’ recommended use of standardised early identification systems to monitor children at risk of becoming critically unwell in hospital [1]. In England, the National Early Warning Score (NEWS2) has been extensively validated for use in adult patients, endorsed by the Royal College of Physicians and is used by the majority of NHS Trusts [2, 3]. The communication of NEWS2 is an adjunct to clinical assessments across England and standardised the process of recognising and responding to the deteriorating adult in hospital [4, 5]. The Royal College of Paediatrics and Child Health and Royal College of Nursing are currently developing a National PEWS for England, with plan for national roll-out [6].

To date, the EPOCH (Evaluating Processes of Care and the Outcomes of Children in Hospital) trial remains the largest and most extensive study of PEWS performance. EPOCH was a prospective cluster randomised cohort study of twenty-one hospital sites across seven countries to evaluate the performance of the Bedside PEWS. No statistically significant difference in all-cause mortality for hospitalised children was reported between sites randomised to Bedside PEWS and standard care [7]. However, in hindsight, it is acknowledged that mortality may not be an appropriate outcome measure in hospitalised children because of low incidence [8].

Morbidity following emergency transfer to critical care following an in-patient deterioration appears to be significant [9]. Critical Deterioration Events (CDE), defined by Bonafide et al. [10] as ‘patients requiring unplanned admission to a critical care area (Paediatric High Dependency or Intensive Care Units) and initiation of organ support within the subsequent 12 hours’, have been suggested as an appropriate metric to evaluate the effectiveness of Rapid Response teams. CDEs occurred 8 times more frequently than cardiac and respiratory arrest, and were associated with a > 13-fold increased risk of in-hospital death [10].

Children with complex medical histories may have physiologically different vital signs than the otherwise healthy child, leading to concerns that a standardised PEWS may be less sensitive for sub-specialties who have a higher risk of deterioration [11, 12]. This challenge was highlighted as an area for research focus by the International Liaison Committee on Resuscitation Paediatric Life Support Task Force [13].

We compared the performance of six established PEWS: Alder Hey [14], Bedside [15], Bristol (Davis P, Haines C, personal communication), Irish [16], Newcastle (Lim E, personal communication), Scotland [17] and the proposed National PEWS for England (v3: as of August 2021) (Sefton G, personal communication) to detect children who experienced an in-hospital CDE.

## Methods

The DETECT (Dynamic Electronic Tracking and Escalation to reduce Critical care Transfers) study prospectively collected vital signs data and matched data about in-hospital deterioration and mortality in a UK tertiary children’s hospital [18].

Pre-intervention data were collected from March 2018 to February 2019 for children admitted to ten inpatient wards. Details of the cohort have been previously published [18]. All patients had the core Alder Hey PEWS recorded (respiratory rate, effort of breathing, oxygen saturation, oxygen requirements, heart rate, capillary refill time, nurse concern of deterioration) on the EPR (Electronic Patient Record) (Meditech v6). Temperature was documented but does not contribute to the local PEWS. Observation sets with a score ≥ 3 prompted recording of the extended PEWS (core components plus blood pressure, level of consciousness using the Alert, Voice, Pain, Unresponsive (AVPU) scale, parental concern).

Observations were excluded from analysis if demographic data or components of the core PEWS were missing. Other missing data were assumed to be normal. The EPR permitted free numerical response for blood pressure, heart rate, respiratory rate and oxygen saturations, and free text response for oxygen therapy. Outlying values were manually reviewed by clinicians to distinguish between physiological extremes versus human error in documentation. Alder Hey PEWS was automatically calculated at the point of documentation in the EPR. The other PEWS were calculated in retrospect, using formulae derived from their paper-based charts.

### PEWS modification for analysis

The database was configured to suit calculation of the local PEWS, limited adaptations were required to the Bedside, Bristol, Irish and Newcastle scores. These are based on the modifications made by Romaine et al. [19] and mostly impacted scoring for respiratory distress. Further detail can be found in Supplementary Table 1.

### Matching cases and controls

Data on cases (patients who experienced a CDE) were collected by the study research nurses. For validation, these were limited to the first CDE during the hospital in-patient visit. Documented vital signs for the preceding 25 h, ceasing 1 h preceding the CDE were included in the analysis. This approach, utilised by Parshuram et al., acknowledges that an optimally performing PEWS needs to signal with adequate notice to allow clinicians to intervene [20]. In addition, as the child becomes more unwell, compensation mechanisms such as tachycardia cannot be sustained [21], meaning that vital signs in the 1 h preceding a CDE are likely to be pre-terminal and unsuitable for configuring a prediction model. The maximum PEWS in the 24, 12, 6 and 4 h periods preceding the hour before CDE, or time of discharge, was identified for analysis. The maximum score has been used conventionally in several paediatric severity score derivation and validation studies [19, 22, 23].

Control patients were matched from a 12-month data repository containing 406,300 observations from 17,269 patient visits who did not have a CDE. They were matched 2:1 to cases, using the Alder Hey PEWS age category (0- <1 year, 1- <2 years, 2- <7 years, 7- <13 years, 13 + years) and the attending specialty. If multiple controls were available, the following criteria were applied; age (months), length of stay (time to discharge versus time to CDE, in days), date of hospital admission (initially month, then day of admission).

Sub-group analysis of score performance was conducted in the 3 specialities with the largest number of CDEs; general paediatrics, cardiology with cardiac surgery and respiratory with long-term ventilation. Mortality was reviewed using the in-hospital annual mortality report to ensure no controls were re-admitted within 72 h following discharge and subsequently died. Score performance was compared using AUC (Area Under the Curve) and the optimum cut-off identified at each time point using the Youden J statistic. This defines the optimal cutoff as the point which maximises the sum of the sensitivity and specificity and has been used in a number of Early Warning Score studies [24–26]. Length of admission was stratified against the defined cut-off with survival analysis (Kaplan-Meier).

### Role of the funding source

This trial is funded by the National Institute of Health Research Invention for Innovation (i4i) programme, funder reference II-LA-0216-20002. This study is supported by the NIHR Clinical Research Network North West Coast.

Disclaimer: The views expressed are those of the authors and not necessarily those of the NHS, the NIHR or the Department of Health and Social Care. Neither the Sponsor not the Funder had any role in the study design; collection, management, analysis and interpretation of data; writing of this manuscript or in the decision to submit this manuscript for publication.

## Results

During the 12-month period, 250 CDEs occurred, with a median patient age of 8 months. 54% of CDEs occurred in children < 1-year of age. Further characteristics of the patient cohort are described in Table 1.

**Table 1:** Descriptive statistics of cases and controls

Variable	Case	Control
*N*	250	500
**Length of stay (days): median(range)**	2 (1, 101)	2 (1, 93)
**Age (months): median(range)**	8 (0, 206)	3 (0, 205)
**Age (categorical)**: ***n*(%)**		
0– < 1yr	136 (54·4)	272 (54·4)
1– < 2yrs	21 (8·4)	42 (8·4)
2– < 7yrs	43 (17·2)	86 (17·2)
7– < 13yrs	27 (10·8)	54 (10·8)
13yrs+	22 (8·8)	44 (8·8)
**Speciality**: ***n*(%)**		
General Paediatrics	89 (35·6)	178 (35·6)
Cardiology	59 (23·6)	118 (23·6)
Respiratory & LTV	38 (15·2)	76 (15·2)
Neurology	17 (6·8)	34 (6·8)
Haematology-Oncology	14 (5·6)	28 (5·6)
General Surgery	12 (4·8)	24 (4·8)
ENT	8 (3·2)	16 (3·2)
Gastroenterology	4 (1·6)	8 (1·6)
Neurosurgery	3 (1·2)	6 (1·2)
Orthopaedics	3 (1·2)	6 (1·2)
Nephrology	2 (0·8)	4 (0·8)
Burns & Plastic Surgery	1 (0·4)	2 (0·4)

Review of the full DETECT cohort showed the median time from worst PEWS to event was 5 h, therefore the 6-hour data are reported in this paper. Further analysis for the other time points can be found in Supplementary Table 2.

To determine the discriminatory properties of each PEWS, ROC curves were constructed for each PEWS and area under the curve calculated (Table 2). An area close to 1 indicates that the PEWS can discriminate between those who were likely to experience a CDE and those who were not. All PEWS showed consistently good performance in discriminating between patients, with overlap across confidence intervals seen across each time interval.

The ROC curves suggest that there is very little difference between the 4, 6 and 12-hour times points at discriminating between cases and non-cases. The 24-hour time-point has slightly lower areas, but still suggest an acceptable level of discrimination.

**Table 2:** Performance of maximum PEWS to predict CDE at 24, 12, 6 and 4 h before event

PEWS	Maximum Score: 24 h	Maximum Score: 12 h	Maximum Score: 6 h	Maximum Score: 4 h
	Area under ROC (95% CI)	Area under ROC (95% CI)	Area under ROC (95% CI)	Area under ROC (95% CI)
Alder Hey	0·94 (0·92, 0·96)	0·95 (0·93, 0·97)	0·95 (0·93, 0·97)	0·95 (0·93, 0·96)
Bedside	0·92 (0·90, 0·94)	0·94 (0·92, 0·96)	0·93 (0·91, 0·95)	0·93 (0·91, 0·95)
Bristol	0·91 (0·89, 0·93)	0·93 (0·91, 0·95)	0·92 (0·90, 0·95)	0·93 (0·91, 0·95)
Irish	0·92 (0·90, 0·94)	0·94 (0·93, 0·96)	0·94 (0·92, 0·96)	0·94 (0·93, 0·96)
National	0·90 (0·87, 0·92)	0·92 (0·90, 0·94)	0·91 (0·90, 0·94)	0·92 (0·89, 0·94)
Newcastle	0·93 (0·91, 0·95)	0·95 (0·93, 0·96)	0·94 (0·92, 0·96)	0·94 (0·92, 0·96)
Scottish	0·87 (0·84, 0·89)	0·89 (0·86, 0·91)	0·89 (0·87, 0·90)	0·88 (0·87, 0·92)

The optimum cut-offs for sensitivity and specificity for each PEWS were identified, and the Kappa coefficient (level of chance adjusted agreement), positive (PPV) and negative (NPV) predictive values were calculated (Table 3). Six hours preceding the CDE, the Alder Hey PEWS, with a cut-off equal to 3, had the highest level of agreement, with NPV and PPV of 0·89 and 0·92 respectively. This suggests that if the child is identified as either being at risk or not at risk of a CDE, approximately 90% accuracy can be expected. The remaining six PEWS also displayed good levels of agreement and NPV and PPV values. At six hours, a cut-off score of 5 was defined as optimum for the proposed National PEWS for England, with NPV and PPV of 0·71 and 0·91 respectively.

The levels of agreement, NPV and PPV for the remaining time points are included in Supplementary Table 2.

**Table 3:** Optimum Cut-Off for the seven PEWS 6 h preceding CDE

	Optimum Cut-off	Kappa (SE)	Sensitivity	Specificity	Negative Predictive Value	Positive Predictive Value
**Maximum PEW 6 h**						
Alder Hey	≥ 3	0·80 (0·024)	0·83	0·95	0·89	0·92
Bedside	≥ 4	0·71 (0·027)	0·86	0·87	0·77	0·93
Bristol	≥ 5	0·65 (0·029)	0·85	0·83	0·71	0·92
Irish	≥ 4	0·67 (0·027)	0·91	0·80	0·70	0·95
National	≥ 5	0·64 (0·029)	0·84	0·83	0·71	0·91
Newcastle	≥ 5	0·76 (0·025)	0·86	0·90	0·82	0·93
Scottish	≥ 4	0·62 (0·030)	0·79	0·85	0·72	0·89

**Fig. 1:**
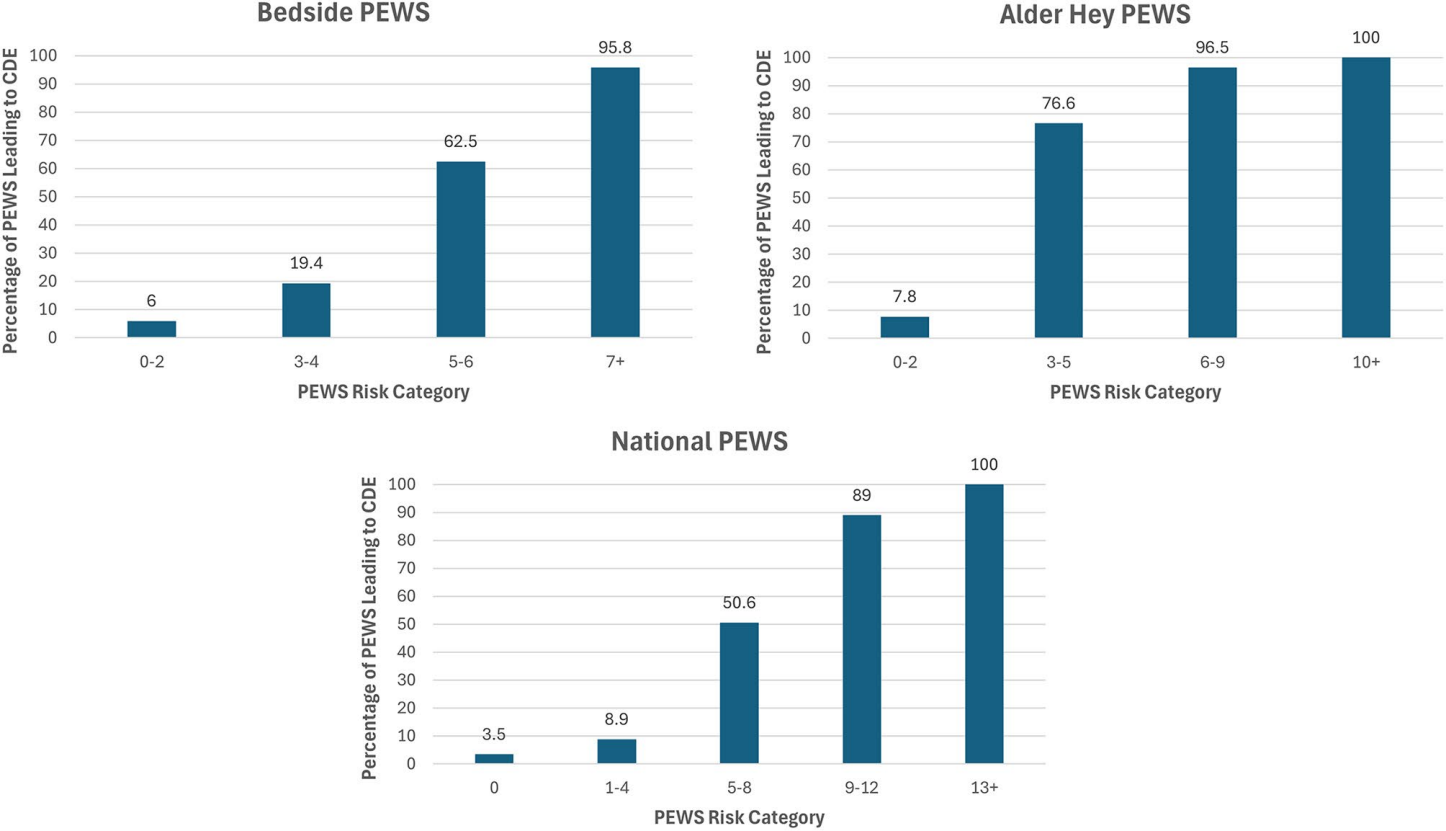
Percentage CDE occurrence within 6 h of worst PEWS in each risk category: Alder Hey PEWS, Bedside PEWS, proposed National PEWS for England.

Each PEWS categorises the calculated scores into risk categories with associated escalation advice. Figure 1 highlights the percentage of observations in each risk category which result in a CDE. For the Alder Hey PEWS, a score of 0–2 is low risk, 3–5 is moderate risk, requiring review by the nurse in charge, 6–9 is high risk and advises urgent medical review within 30 min, 10 + is critical risk, and advises urgent review by a senior clinician within 10 min, or to contact the crash team.

Direct comparison of all seven PEWS is challenging as the risk stratification for deterioration risk and expected local response differ. Therefore, we have focused on a more detailed analysis comparing the local PEWS (Alder Hey) with Bedside (the most studied PEWS) and the National PEWS (prior to National roll-out). The risk categorisation for the Bedside PEWS were derived from a clinical audit by Gawronski et al. reflecting the use of the system at a tertiary children’s hospital in central Italy [27]. The proposed National PEWS is the only one that contains five risk categories, as any score greater than 0 necessitates some level of review. However, the percentage of patients experiencing CDE across the first two categories is low.

Graphs representing the frequency of CDE per score value are reported in Supplementary Fig. 1, along with frequency tables for all 4 timepoints in Supplementary Table 3.

**Fig. 2:**
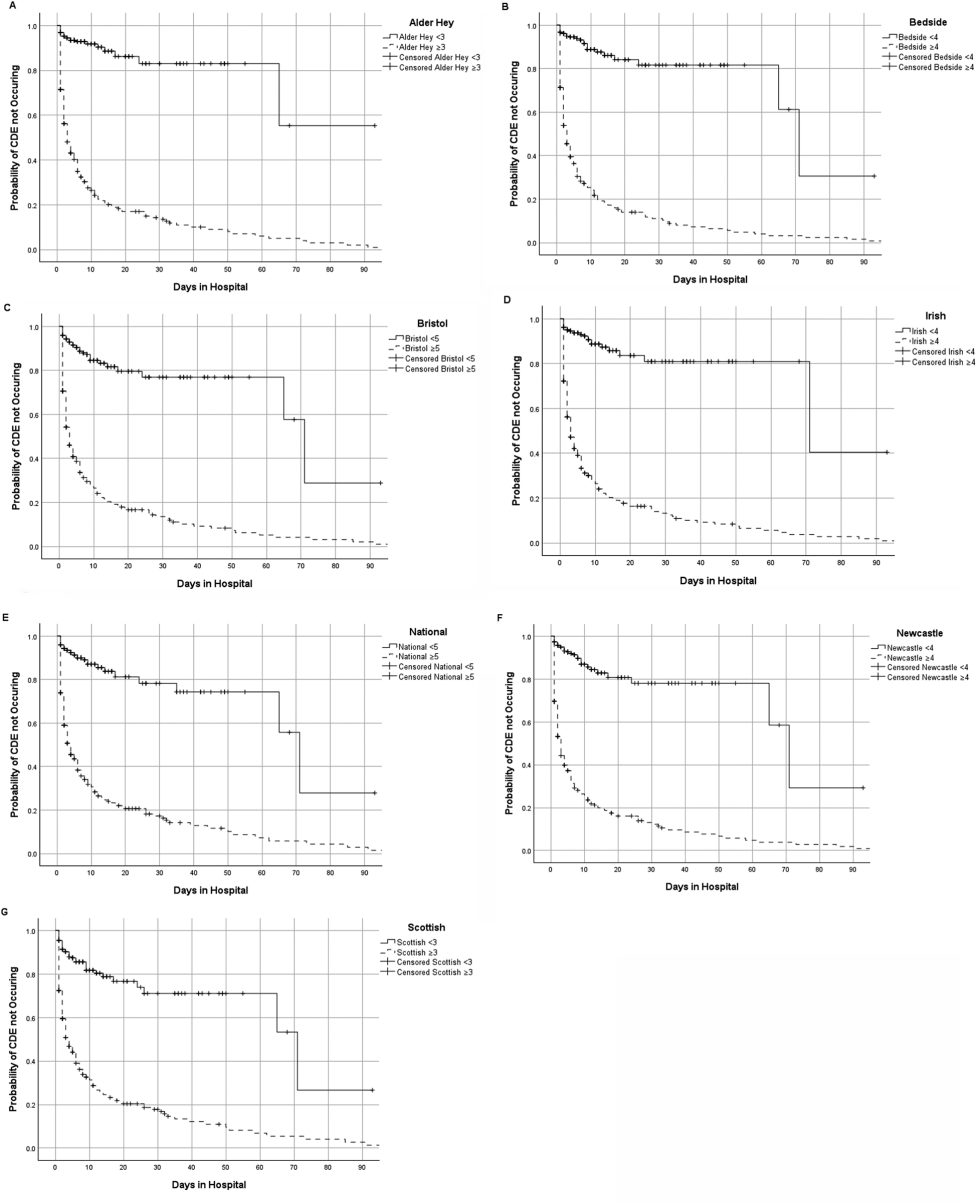
Kaplan-Meier Survival Curves demonstrating cumulative time to event in PEWS above and below the identified optimal cut-points for sensitivity 6 h preceding CDE for **(A)** Alder Hey PEWS, **(B)** Bedside PEWS, **(C)** Bristol PEWS, **(D)** Irish PEWS, **(E)** Proposed National PEWS for England, **(F)** Newcastle PEWS, **(G)** Scottish PEWS

Figure 2 presents Kaplan-Meier curves comparing the cumulative time to event (where hospital discharge is represented as censored data). Data are displayed using the highest PEWS in the preceding 6 h, stratified by the identified optimum cut-off value. The vertical axis represents the cumulative probability a child will survive the period of time on the horizontal axis without having a CDE.

In each PEWS studied, patients above the optimum score were more likely to experience CDE. This was statistically significant for all PEWS (*p* < 0·001). Similar results were obtained for all the other time points, reported in Supplementary Fig. 2.

**Table 4:** Performance of maximum PEWS to predict CDE at 6 h before event in sub-specialty patients

PEW	General Paediatrics (35·6% of cohort)	Cardiology (23·6% of cohort)	Respiratory (15·2% of cohort)
	Area under ROC (95% CI)	Area under ROC (95% CI)	Area under ROC (95% CI)
Alder Hey	0·98 (0·96, 0·99)	0·93 (0·89, 0·97)	0·94 (0·89, 0·99)
Bedside	0·97 (0·94, 0·99)	0·89 (0·83, 0·95)	0·93 (0·88, 0·98)
Bristol	0·97 (0·94, 0·99)	0·87 (0·81, 0·93)	0·91 (0·86, 0·97)
Irish	0·97 (0·95, 0·99)	0·90 (0·85, 0·95)	0·94 (0·89, 0·99)
National	0·96 (0·93, 0·98)	0·82 (0·90, 0·94)	0·91 (0·85, 0·97)
Newcastle	0·98 (0·96, 0·99)	0·91 (0·86, 0·96)	0·93 (0·87, 0·99)
Scottish	0·92 (0·88, 0·95)	0·87 (0·82, 0·92)	0·86 (0·79, 0·93)

Table 4 presents the sub-group ROC analysis for cardiology, respiratory and general paediatrics. The predictive performance of each PEWS remains very good, with overlapping confidence intervals within each PEWS. The marginal reduction in the AUC for the cardiology sub-specialty is still considered excellent [28].

**Table 5:** Performance of maximum PEWS to identify 72-hour mortality from maximum PEWS in all patients

PEWS	Maximum Score: 24 h	Maximum Score: 12 h	Maximum Score: 6 h	Maximum Score: 4 h
	Area under ROC (95% CI)	Area under ROC (95% CI)	Area under ROC (95% CI)	Area under ROC (95% CI)
Alder Hey	0·58 (0·36–0·81)	0·82 (0·72–0·92)	0·85 (0·74–0·95)	0·88 (0·76–0·98)
Bedside	0·50 (0·28–0·73)	0·79 (0·70–0·88)	0·77 (0·67–0·96)	0·79 (0·69–0·89)
Bristol	0·59 (0·39–0·78)	0·83 (0·76–0·90)	0·84 (0·76–0·91)	0·83 (0·71–0·95)
Irish	0·64 (0·40–0·89)	0·84 (0·74–0·94)	0·82 (0·73–0·92)	0·83 (0·71–0·95)
National	0·56 (0·34–0·79)	0·80 (0·70–0·90)	0·79 (0·70–0·89)	0·81 (0·70–0·92)
Newcastle	0·61 (0·42–0·79)	0·82 (0·75–0·90)	0·84 (0·76–0·92)	0·83 (0·71–0·94)
Scottish	0·74 (0·57–0·91)	0·90 (0·84–0·95)	0·90 (0·85–0·96)	0·86 (0·75–0·97)

Table 5 presents the ROC analysis for 72-hour mortality. In the whole cohort, performance ranges between 0·90 for the Scottish PEWS and 0·50 for the Bedside PEWS, with much wider confidence intervals. The results suggest that the 24-hour AUCs are poor, but the 12, 6, 4-hour AUCs show reasonable levels of discrimination. However, as there were only 6 deaths, these results should be treated with caution.

When comparing the maximum PEWS in the preceding 24-hours, all but the Scottish PEWS are significantly better at predicting CDE than mortality. As mortality is a rare outcome in paediatrics, the performance of the score may be proportional to the frequency of the outcome [29].

## Discussion

This is the first study to compare the predictive performance of various PEWS used in the United Kingdom and Ireland for identifying CDEs in hospitalised children and is a retrospective database evaluation of the proposed National PEWS for England. The 7 PEWS evaluated represent those most used in the UK and Ireland, and all demonstrated excellent prediction for CDEs across the four timepoints. The National PEWS performed as well as the other scores in use, with no significant differences in performance. We have demonstrated that a single risk model is applicable across high-risk sub-specialty groups as well as in a general cohort. This is a strong case for the standardisation using the National PEWS for England.

Within our cohort there is some difference in AUC over the four time points, albeit with overlap in confidence intervals. As PEWS is a dynamic assessment of physiological clinical state, it is not unexpected that the AUROC would alter as a patient progresses towards a pre-terminal event. When compared to other literature, there is some observed difference in AUC performance. Chapman has reported AUC for 19 PEWS but used the maximum observed value for each PEWS for each patient in the 47 h preceding event [8]. They reported AUC of 0.88 for Bedside (0.85–0.91) and 0.62 (0.58–0.67) for Bristol. Parshuram has previously reported AUC for the Bedside PEWS of 0.91 in its identification of patients who were urgently admitted to PICU (but excluding those admitted following a call for immediate medical assistance) with data collected over a 12-hour period in case-patients and 24-hours in controls [30]. These differences may reflect the different patient populations included in each study, with our patient population representing a mixture of general paediatric admissions along with tertiary subspecialty care. Our observation utilised maximum score performance within a set period, as maximum score plays a significant role in escalation of the child for medical review.

Some variation is seen within the optimum cut-off analysis for every score except the Alder Hey (locally calibrated) PEWS. In each of the other six PEWS, the optimum cut-off analysis is lower in the maximum PEWS 4-hours preceding the event than 24-hours before. One possibility is that as patients develop decompensated shock, their observations may falsely normalise (tachycardia and tachypnoea may settle for example). This could be mis-interpreted as a normalisation of the PEWS and emphasises the importance of clinical review of the child. Instead of the PEWS signalling an imminent, preventable deterioration (which is not always the case), a child who is ‘scoring’ should act as a prompt for focused situational awareness, to acknowledge response to treatment and consider if the course of deterioration could be avoided. Here the cut-off analysis was data-driven, rather than developed by consensus as was the case in the development of many PEWS. The six-hour PEWS was reported as this was closest to the median time to CDE from maximum PEWS reported in our cohort. The aim of the PEWS is to minimise unexpected deteriorations and allow preparedness where possible, as most CDEs have a level of forewarning. The performance at earlier and later time points was evaluated to assess the performance of these prompts at different time intervals in the 24 h preceding deterioration. The use of data-informed cut-points aims to balance preparedness with alarm fatigue.

When comparing scores, it is important to consider that this was a prospective evaluation of the Alder Hey score, and a retrospective database evaluation of the other six, and that the surrounding early warning system (for example the threshold for response) for each of these scores was not evaluated as that is outside of the scope of this piece of work. The score is one piece of the organisational process to identify and manage deterioration in hospitalised children. This work illustrates that an aggregate PEWS score can predict the occurrence of critical deterioration, but this is futile without a rapid response system allowing clinical intervention in these cases. Work from the PUMA Programme (Paediatric early warning system– Utilisation and Mortality Avoidance improvement programme) has recognised this and is developing metrics to assess the performance of paediatric early warning systems [31]. The PEW Systems may also have a built in “fail safe”, aiming to identify deteriorating children which the model may not take account of. An example of this ‘real-life’ system override within this study is the Scottish PEWS, where professional concern overrides the score, prompting an urgent clinical review, regardless of the PEWS. Families can contact their named nurse, nurse-in-charge, co-ordinator or, in some cases, doctor or outreach team to raise their concerns [32].

General Paediatrics, Cardiology and Respiratory specialties had the highest number of CDEs. As described in Supplementary Table 4, almost one-third of patients were admissions under General Paediatrics, which is likely to explain why 35% of the CDEs occurred in this group. Cardiology and Respiratory made up 6% and 3·3% of admissions but had 23% and 15·2% of CDEs respectively. Since those patients are at higher risk of inpatient deterioration; this suggests monitoring would be appropriate to mitigate risk, allow for timely identification of deterioration progressing and early goal-directed interventions. There has been suggestion that a generalised PEWS would not be appropriate for these patients. For example, a cardiac-specific PEWS may account for hypoxia relative to the patient baseline rather than at fixed saturations [33, 34], compared to a ‘standard’ chronically elevated PEWS reflecting altered physiology in cyanotic heart defects where staff have made clinical judgements the child is not deteriorating. This variation in PEWS utilisation has been described in the acknowledged variation in AUC when PEWS have been evaluated in retrospective datasets with the assumption that PEWS will be escalated as per algorithm, compared with prospective evaluation studies [20, 30]. Our evaluation found that PEWS predictive performance remained very good within these cohorts and suggests a standardised PEWS is generalisable to all paediatric ward in-patients, including patients with asthma, bronchiolitis and with cardiology diagnoses.

EPOCH used mortality as the primary outcome to evaluate the effectiveness of the Bedside PEWS. Mortality is a rare event in paediatrics, and despite the international study the observed mortality in the intervention and comparator arm were significantly better than the power calculations used for study design. Paediatric critical care mortality has decreased over time which was also acknowledged in PICANet data [35]. Observed hospital mortality was low in this cohort, with death within 72-hours following CDE occurring in six patients, despite the study utilising 12 months of data from a tertiary children’s hospital. In this data repository CDEs occurred 18 times more frequently than in-hospital deaths and are a significant cause of morbidity [33, 36]. Bonafide reported a 13-fold increased risk of mortality following CDE [10]. This has been highlighted in other bodies of work studying the efficacy and performance value of PEWS [8], and is a key distinguishing factor between a paediatric and adult-oriented early warning score. Our work suggests that in paediatrics CDE is a more appropriate outcome marker, although it is more difficult to quantify.

### Strengths

We have compared the predictive performance of PEWS in active use across the United Kingdom and Ireland, in a rigorously collected large heterogenous dataset of hospitalised children, and the subsequent prevalence of CDE. Differentiating the hospital in-patients who did and did not have a CDE is a clinically appropriate outcome to evaluate, with potential to improve clinical outcomes and the safety cultures for hospital in-patients.

Although this was a single centre study, the hospital provides general paediatric services for the local population, as well as tertiary and quaternary care across the region. The findings are therefore applicable to both local, ‘district general hospital’ settings and larger, specialist services.

### Limitations

Data were collected prospectively for the DETECT study, by a dedicated team, including the AH PEWS, but evaluation of the other PEWS performance was retrospective. In particular, the assessment of the respiratory distress criteria within each PEWS were limited by the data collected in the database. Therefore, clinical decision and experience had to be applied to the interpretation of those variables (Supplementary Table 1).

The three sub-specialities which experienced the most CDEs underwent a further analysis of score performance. Haematology-Oncology is acknowledged as a high-risk specialty with patient with increased risk of CDE related to sepsis or chemotherapy related organ dysfunction [37–39], and had been identified as a potential risk group where focused evaluation of PEWS performance could benefit care. However, the observed number of cases was too small for meaningful sub-analysis (*n* = 14). Neurology also had a higher percentage of CDEs (6·8% of total, 5·17% of patients admitted) but again there were insufficient cases (*n* = 17).

Patients who experienced a CDE were not reintroduced to the dataset within that admission, as it is unclear how long it takes for a patient to return to acceptable physiological values post CDE. The study therefore does not assess subsequent CDEs within the same admission, which have the potential of being higher-risk events [40].

## Conclusions

All seven PEWS assessed demonstrate excellent predictive ability for CDE, in a heterogenous cohort. For evaluation of PEWS performance, CDE is a more appropriate outcome measure than hospital mortality, due to low mortality outside PICU. We have demonstrated that a single risk model is applicable across high-risk sub-specialty groups as well as in a general cohort. Our study makes a strong case for the standardisation using the National PEWS for England and would encourage standardisation of the corresponding paediatric early warning system. A national standardised PEWS would allow collation of big data across primary to tertiary units to develop evidence-based thresholds for children admitted to hospital, modelling for weighting of PEWS components and the opportunity for periodic recalibration of age-specific risk models.

Standardisation of PEWS creates a “common language” across different settings and aids benchmarking of practice. This single centre study supports the widespread roll out of proposed National PEWS for England, for predicting the occurrence of CDEs in hospitalised children. Further validation is required in other settings to further enhance the available evidence base.

## Electronic supplementary material

Below is the link to the electronic supplementary material.

Supplementary Material 1

## Data Availability

The datasets generated and analysed during the current study are not publicly available due to the data consisting of a small number of cases with critical deterioration event occurrence. In a single centre study including several children with co-morbidities and frequent hospitalisations, data sharing would risk identification of participants. Datasets are available from the corresponding author on reasonable request.

## Declarations

Vital signs documentation and sepsis screening form part of routine clinical care and consent was not required for the devices to record these changes. Posters outlining the purpose of the DETECT study were displayed in in-patient wards, giving permission to parents to decline the use of their child’s anonymised vital signs data in analysis. Informed consent was collected for families of children transferred to critical care, including statements about the use of anonymised data for publication as stated in the DETECT study protocol paper^18^.

## Abbreviations

NEWS: National Early Warning Score
PEWS: Paediatric Early Warning Score
CDE: Critical Deterioration Event
DETECT: Dynamic Electronic Tracking and Escalation to reduce Critical care Transfers
EPR: Electronic Patient Record
AUC: Area Under the Curve
ROC: Receiver Operating Curves
PPV: Positive Predictive Value
NPV: Negative Predictive Value
PEW: Paediatric Early Warning

## References

[1] Pearson GA. Why Children Die: A Pilot Study 2006; England (South West, North East and West Midlands), Wales and Northern Ireland. London; 2008.

[2] Physicians RCo. *National Early Warning Score (NEWS) 2: Standardising the assessment of acute-illness severity in the NHS.* Updated report of a working party. London: RCP; 2017.

[3] Smith GB, Prytherch DR, Meredith P, Schmidt PE, Featherstone PI. The ability of the National early warning score (NEWS) to discriminate patients at risk of early cardiac arrest, unanticipated intensive care unit admission, and death. Resuscitation. 2013;84(4):465–70. 23295778 10.1016/j.resuscitation.2012.12.016

[4] Inada-Kim M, Nsutebu E. NEWS 2: an opportunity to standardise the management of deterioration and sepsis. BMJ. 2018;360:k1260. 29559439 10.1136/bmj.k1260

[5] Inada-Kim M, Knight T, Sullivan M, Ainsworth-Smith M, Pike N, Richardson M et al. The prognostic value of National early warning scores (NEWS) during transfer of care from community settings to hospital: a retrospective service evaluation. BJGP Open. 2020 Jun 23;4(2). 10.3399/bjgpopen20X101071 PMC7330211 32398345

[6] Roland D, Stilwell PA, Fortune PM, Alexander J, Clark SJ, Kenny S. Case for change: a standardised inpatient paediatric early warning system in England. Arch Dis Child. 2021;106(7):648–51. 33419727 10.1136/archdischild-2020-320466

[7] Parshuram CS, Dryden-Palmer K, Farrell C, Gottesman R, Gray M, Hutchison JS, et al. Effect of a pediatric early warning system on All-Cause mortality in hospitalized pediatric patients: the EPOCH randomized clinical trial. JAMA. 2018;319(10):1002–12. 29486493 10.1001/jama.2018.0948 PMC5885881

[8] Chapman SM, Wray J, Oulton K, Peters MJ. Death is not the answer’: the challenge of measuring the impact of early warning systems. Arch Dis Child. 2019;104(3):210–1. 30217860 10.1136/archdischild-2018-315392

[9] Trubey R, Huang C, Lugg-Widger FV, Hood K, Allen D, Edwards D, et al. Validity and effectiveness of paediatric early warning systems and track and trigger tools for identifying and reducing clinical deterioration in hospitalised children: a systematic review. BMJ Open. 2019;9(5):e022105. 31061010 10.1136/bmjopen-2018-022105 PMC6502038

[10] Bonafide CP, Roberts KE, Priestley MA, Tibbetts KM, Huang E, Nadkarni VM, et al. Development of a pragmatic measure for evaluating and optimizing rapid response systems. Pediatrics. 2012;129(4):e874–81. 22392182 10.1542/peds.2011-2784

[11] Dean NP, Fenix JB, Spaeder M, Levin A. Evaluation of a pediatric early warning score across different subspecialty patients. Pediatr Crit Care Med. 2017;18(7):655–60. 28445240 10.1097/PCC.0000000000001176

[12] Dewan M, Tegtmeyer K. Do subspecialty patients need special evaluation to screen for deterioration?? Pediatr Crit Care Med. 2017;18(7):723–4. 28691963 10.1097/PCC.0000000000001184

[13] Chong SL, Goh MSL, Ong GY, Acworth J, Sultana R, Yao SHW, et al. Do paediatric early warning systems reduce mortality and critical deterioration events among children? A systematic review and meta-analysis. Resusc Plus. 2022;11:100262. 35801231 10.1016/j.resplu.2022.100262 PMC9253845

[14] Sefton G, Lane S, Killen R, Black S, Lyon M, Ampah P, et al. Accuracy and efficiency of recording pediatric early warning scores using an electronic physiological surveillance system compared with traditional Paper-Based Documentation. Comput Inf Nurs. 2017;35(5):228–36. 10.1097/CIN.0000000000000305 PMC5708717 27832032

[15] Parshuram CS, Bayliss A, Reimer J, Middaugh K, Blanchard N. Implementing the bedside paediatric early warning system in a community hospital: A prospective observational study. Paediatr Child Health. 2011;16(3):e18–22. 22379384 10.1093/pch/16.3.e18 PMC3077313

[16] Department of Health. (2016 V. The Irish Paediatric Early Warning System (PEWS) (NCEC National Clinical Guideline No. 12). 2016.

[17] Scotland HI. Paediatric Early Warning Score (PEWS) Charts Scotland: The Improvement Hub; 2017 [Available from: https://ihub.scot/improvement-programmes/scottish-patient-safety-programme-spsp/spsp-programmes-of-work/maternity-and-children-quality-improvement-collaborative-mcqic/paediatric-care/pews/

[18] Sefton G, Carter B, Lane S, Peak M, Mateus C, Preston J, et al. Dynamic electronic tracking and escalation to reduce critical care transfers (DETECT): the protocol for a stepped wedge mixed method study to explore the clinical effectiveness, clinical utility and cost-effectiveness of an electronic physiological surveillance system for use in children. BMC Pediatr. 2019;19(1):359. 31623583 10.1186/s12887-019-1745-7 PMC6796473

[19] Romaine ST, Sefton G, Lim E, Nijman RG, Bernatoniene J, Clark S, et al. Performance of seven different paediatric early warning scores to predict critical care admission in febrile children presenting to the emergency department: a retrospective cohort study. BMJ Open. 2021;11(5):e044091. 33947731 10.1136/bmjopen-2020-044091 PMC8098996

[20] Parshuram CS, Duncan HP, Joffe AR, Farrell CA, Lacroix JR, Middaugh KL, et al. Multicentre validation of the bedside paediatric early warning system score: a severity of illness score to detect evolving critical illness in hospitalised children. Crit Care. 2011;15(4):R184. 21812993 10.1186/cc10337 PMC3387627

[21] Sinha R, Nadel S. Understanding shock. Paediatrics Child Health. 2013;23(5):187–93.

[22] Matics TJ, Sanchez-Pinto LN. Adaptation and validation of a pediatric sequential organ failure assessment score and evaluation of the Sepsis-3 definitions in critically ill children. JAMA Pediatr. 2017;171(10):e172352. 28783810 10.1001/jamapediatrics.2017.2352 PMC6583375

[23] Schlapbach LJ, Straney L, Bellomo R, MacLaren G, Pilcher D. Prognostic accuracy of age-adapted SOFA, SIRS, PELOD-2, and qSOFA for in-hospital mortality among children with suspected infection admitted to the intensive care unit. Intensive Care Med. 2018;44(2):179–88. 29256116 10.1007/s00134-017-5021-8 PMC5816088

[24] Ozdemir S, Akca HS, Algin A, Altunok I, Eroglu SE. Effectiveness of the rapid emergency medicine score and the rapid acute physiology score in prognosticating mortality in patients presenting to the emergency department with COVID-19 symptoms. Am J Emerg Med. 2021;49:259–64. 34171720 10.1016/j.ajem.2021.06.020 PMC8191303

[25] Wang F, An W, Zhang X. Copeptin combined with National early warning score for predicting survival in elderly critical ill patients at emergency department. Am J Emerg Med. 2021;49:153–7. 34116468 10.1016/j.ajem.2021.05.052

[26] Bayer O, Schwarzkopf D, Stumme C, Stacke A, Hartog CS, Hohenstein C, et al. An early warning scoring system to identify septic patients in the prehospital setting: the PRESEP score. Acad Emerg Med. 2015;22(7):868–71. 26113162 10.1111/acem.12707

[27] Gawronski O, Ferro F, Cecchetti C, Ciofi Degli Atti M, Dall’Oglio I, Tiozzo E, et al. Adherence to the bedside paediatric early warning system (BedsidePEWS) in a pediatric tertiary care hospital. BMC Health Serv Res. 2021;21(1):852. 34419038 10.1186/s12913-021-06809-2 PMC8380378

[28] Mandrekar JN. Receiver operating characteristic curve in diagnostic test assessment. J Thorac Oncol. 2010;5(9):1315–6. 20736804 10.1097/JTO.0b013e3181ec173d

[29] Romero-Brufau S, Huddleston JM, Escobar GJ, Liebow M. Why the C-statistic is not informative to evaluate early warning scores and what metrics to use. Crit Care. 2015;19:285. 26268570 10.1186/s13054-015-0999-1 PMC4535737

[30] Parshuram CS, Hutchison J, Middaugh K. Development and initial validation of the bedside paediatric early warning system score. Crit Care. 2009;13(4):R135. 19678924 10.1186/cc7998 PMC2750193

[31] Roland D, Powell C, Lloyd A, Trubey R, Tume L, Sefton G et al. Paediatric early warning systems: not a simple answer to a complex question. Arch Dis Child. 2022 Jul 22;108(5):338–343. 10.1136/archdischild-2022-323951 PMC10176370 35868852

[32] Brady PW, Zix J, Brilli R, Wheeler DS, Griffith K, Giaccone MJ, et al. Developing and evaluating the success of a family activated medical emergency team: a quality improvement report. BMJ Qual Saf. 2015;24(3):203–11. 25516987 10.1136/bmjqs-2014-003001

[33] Lambert V, Matthews A, MacDonell R, Fitzsimons J. Paediatric early warning systems for detecting and responding to clinical deterioration in children: a systematic review. BMJ Open. 2017;7(3):e014497. 28289051 10.1136/bmjopen-2016-014497 PMC5353324

[34] McLellan MC, Gauvreau K, Connor JA. Validation of the cardiac children’s hospital early warning score: an early warning scoring tool to prevent cardiopulmonary arrests in children with heart disease. Congenit Heart Dis. 2014;9(3):194–202. 23957443 10.1111/chd.12132

[35] Universities of Leeds and Leicester. PICANet: A Decade of Data. 2014 September 2014.

[36] Agulnik A, Mora Robles LN, Forbes PW, Soberanis Vasquez DJ, Mack R, Antillon-Klussmann F, et al. Improved outcomes after successful implementation of a pediatric early warning system (PEWS) in a resource-limited pediatric oncology hospital. Cancer. 2017;123(15):2965–74. 28440868 10.1002/cncr.30664

[37] Skaletzky SM, Raszynski A, Totapally BR. Validation of a modified pediatric early warning system score: a retrospective case-control study. Clin Pediatr (Phila). 2012;51(5):431–5. 22157421 10.1177/0009922811430342

[38] Tibballs J, Kinney S. Reduction of hospital mortality and of preventable cardiac arrest and death on introduction of a pediatric medical emergency team. Pediatr Crit Care Med. 2009;10(3):306–12. 19307806 10.1097/PCC.0b013e318198b02c

[39] Tume L. The deterioration of children in ward areas in a specialist children’s hospital. Nurs Crit Care. 2007;12(1):12–9. 17883659 10.1111/j.1478-5153.2006.00195.x

[40] Odetola FO, Clark SJ, Dechert RE, Shanley TP. Going back for more: an evaluation of clinical outcomes and characteristics of readmissions to a pediatric intensive care unit. Pediatr Crit Care Med. 2007;8(4):343–7. CEU quiz 57. 17545926 10.1097/01.PCC.0000269400.67463.AC

