# Supplementary material for: Assessing the performance of paediatric early warning scores to predict critical deterioration events in hospitalised children (the DETECT study): a retrospective matched case-control study
Source: BMC Pediatr. 2025 Jul 2;25:520. doi: 10.1186/s12887-025-05754-x (PMC12220617; doi:10.1186/s12887-025-05754-x)
Supplement: Supplementary file 1 — Supplementary Material 1 [file 12887_2025_5754_MOESM1_ESM.docx]

**Assessing the performance of paediatric early warning scores to predict CDE in hospitalised children (the DETECT study): a retrospective matched case-control study - Supplementary:**

**Suppl. Table 1: Component Breakdown of the Seven Paediatric Early Warning Scores**

| **PEWS** | **AVPU** | **Capillary Refill Time** | **Heart Rate** | **Carer Concern** | **Nursing/Clinician Concern** | **Oxygen**  **Therapy** | **Oxygen Delivery** | **Respiratory Distress** | **Respiratory Rate** | **Oxygen Saturation** | **Systolic Blood Pressure** | **Temperature** |
| --- | --- | --- | --- | --- | --- | --- | --- | --- | --- | --- | --- | --- |
| **Alder Hey** | ✓ | ✓ | ✓ | ✓ | ✓ | ✓ | × | ✓ | ✓ | ✓ | ✓ | × |
| **Bedside** | × | ✓ | ✓ | × | × | ✓ | × |  | ✓ | ✓ | ✓ | × |
| **Bristol** | ✓ | ✓ | ✓ | × | × | ✓ | ✓ |  | ✓ | ✓ | ✓ | ✓ |
| **Irish** | ✓ | ✓ | ✓ | ✓ | ✓ | ✓ | ✓ |  | ✓ | ✓ | ✓ | × |
| **National** | × | ✓ | ✓ | × | × | ✓ | × |  | ✓ | ✓ | ✓ | × |
| **Newcastle** | ✓ | ✓ | ✓ | ✓ | ✓ | ✓ | × |  | ✓ | ✓ | ✓ | ✓ |
| **Scotland** | ✓ | ✓ | ✓ | × | × | ✓ | × | × | ✓ | ✓ | ✓ | ✓ |

Key:

| ✓ - used as intended | × - not included in score | - - used with modification |
| --- | --- | --- |

Respiratory Distress:

The Bedside and Bristol PEWS do not give criteria for assessing the level of respiratory distress, rather marking it as mild, moderate or severe. Instead, the criteria for the National PEWS were used for this scoring.
The Irish PEWS includes a grading criterion for severity of stridor – marking it as mild, moderate or severe. This information was not collected at the time of observation, so any patient with stridor was marked as moderate distress (consistent with the National/Newcastle PEWS severity for inspiratory noises).

As it is not included in the Alder Hey PEWS scoring criteria, data was not collected about the presence of: exhaustion, impending respiratory arrest and tripoding, so these criteria could not be scored for.

Data was collected about the presence of either mild recession or marked subcostal recession. This study used the same approach as *Romaine et al* and scored mild recession as a 'mild' criterion and marked subcostal recession as a 'severe' criterion. We were therefore unable to score for intercostal recession as a 'moderate' criterion.

**Suppl. Table 2: Optimum Cut-Offs for the Seven Paediatric Early Warning Scores at 24, 12, 6 and 4 Hours**

|  | Optimum Cut-off | Kappa(se) | Sensitivity | Specificity | Negative Predictive Value | Positive Predictive Value |
| --- | --- | --- | --- | --- | --- | --- |
| **Maximum PEW 4 hours** | | | | | | |
| Alder Hey | ≥3 | 0.77 (0.025) | 0.79 | 0.96 | 0.90 | 0.90 |
| Bedside | ≥4 | 0.70 (0.027) | 0.82 | 0.89 | 0.79 | 0.93 |
| Bristol | ≥5 | 0.65 (0.029) | 0.80 | 0.86 | 0.74 | 0.90 |
| Irish | ≥5 | 0.74 (0.026) | 0.84 | 0.91 | 0.82 | 0.92 |
| National | ≥5 | 0.66 (0.029) | 0.80 | 0.86 | 0.75 | 0.90 |
| Newcastle | ≥4 | 0.75 (0.026) | 0.81 | 0.93 | 0.85 | 0.91 |
| Scottish | ≥3 | 0.52 (0.032) | 0.79 | 0.77 | 0.63 | 0.88 |
| **Maximum PEW 6 hours** | | | | | | |
| Alder Hey | ≥3 | 0.80 (0.024) | 0.83 | 0.95 | 0.89 | 0.92 |
| Bedside | ≥4 | 0.71 (0.027) | 0.86 | 0.87 | 0.77 | 0.93 |
| Bristol | ≥5 | 0.65 (0.029) | 0.85 | 0.83 | 0.71 | 0.92 |
| Irish | ≥4 | 0.67 (0.027) | 0.91 | 0.80 | 0.70 | 0.95 |
| National | ≥5 | 0.64 (0.029) | 0.84 | 0.83 | 0.71 | 0.91 |
| Newcastle | ≥5 | 0.76 (0.025) | 0.86 | 0.90 | 0.82 | 0.93 |
| Scottish | ≥4 | 0.62 (0.030) | 0.79 | 0.85 | 0.72 | 0.89 |
| **Maximum PEW 12 hours** | | | | | | |
| Alder Hey | ≥3 | 0.80 (0.024) | 0.80 | 0.97 | 0.92 | 0.91 |
| Bedside | ≥4 | 0.76 (0.026) | 0.86 | 0.93 | 0.91 | 0.93 |
| Bristol | ≥6 | 0.71 (0.028) | 0.68 | 0.98 | 0.94 | 0.86 |
| Irish | ≥5 | 0.76 (0.025) | 0.85 | 0.91 | 0.83 | 0.92 |
| National | ≥6 | 0.68 (0.029) | 0.74 | 0.92 | 0.83 | 0.88 |
| Newcastle | ≥6 | 0.77 (0.025) | 0.78 | 0.90 | 0.96 | 0.90 |
| Scottish | ≥5 | 0.56 (0.033) | 0.57 | 0.90 | 0.84 | 0.81 |
| **Maximum PEW 24 hours** | | | | | | |
| Alder Hey | ≥3 | 0.70 (0.027) | 0.88 | 0.85 | 0.75 | 0.93 |
| Bedside | ≥5 | 0.69 (0.027) | 0.86 | 0.85 | 0.75 | 0.93 |
| Bristol | ≥7 | 0.67 (0.029) | 0.81 | 0.87 | 0.76 | 0.90 |
| Irish | ≥6 | 0.68 (0.028) | 0.86 | 0.85 | 0.77 | 0.92 |
| National | ≥6 | 0.61 (0.030) | 0.84 | 0.80 | 0.68 | 0.91 |
| Newcastle | ≥6 | 0.69 (0.028) | 0.84 | 0.86 | 0.76 | 0.92 |
| Scottish | ≥5 | 0.48 (0.030) | 0.88 | 0.67 | 0.57 | 0.87 |

**Suppl. Table 3: Frequency of CDEs per PEW score**

Suppl. Table 3A: **Maximum 4-hour PEW**

| PEW score | Alder Hey | Bedside | Bristol | Irish | National | Newcastle | Scottish |
| --- | --- | --- | --- | --- | --- | --- | --- |
| 0 | 7/323 (2.2) | 4/115 (3.5) | 3/109 (2.8) | 3/128 (2.3) | 2/72 (4.2) | 3/136 (2.2) | 5/174 (2.9) |
| 1 | 16/108 (14.9) | 8/175 (4.6) | 6/131 (4.9) | 4/149 (2.7) | 6/142 (4.2) | 6/172 (3.5) | 14/178 (7.9) |
| 2 | 21/91 (23.1) | 11/128 (8.6) | 6/121 (5.0) | 6/98 (6.1) | 10/148 (6.8) | 8/99 (8.1) | 25/76 (32.9) |
| 3 | 17/33 (51.6) | 13/63 (20.6) | 10/56 (17.9) | 8/58 (13.8) | 8/65 (12.3) | 12/57 (21.1) | 13/67 (19.4) |
| 4 | 20/24 (83.3) | 12/41 (29.4) | 15/53 (28.3) | 10/51 (19.6) | 13/45 (28.9) | 9/37 (24.3) | 37/68 (54.4) |
| 5 | 30/30 (100) | 15/29 (51.7) | 8/39 (20.5) | 17/37 (46.9) | 16/46 (34.8) | 16/31 (51.6) | 32/47 (68.1) |
| 6 | 30/31 (96.3) | 30/37 (81.1) | 14/33 (42.4) | 13/24 (54.2) | 21/41 (51.2) | 17/25 (68.0) | 27/37 (73.0) |
| 7 | 27/28 (96.8) | 15/19 (83.3) | 16/22 (72.7) | 23/28 (82.1) | 17/24 (70.8) | 15/22 (68.2) | 23/27 (85.2) |
| 8 | 22/22 (100) | 14/15 (93.3) | 25/32 (78.1) | 33/41 (80.5) | 21/24 (87.5) | 14/17 (82.4) | 33/35 (94.3) |
| 9 | 23/23 (100) | 39/39 (100) | 16/20 (80.0) | 33/34 (97.1) | 17/22 (77.3) | 19/21 (90.5) | 14/14 (100) |
| 10+ | 28/28 (100) | 86/87 (99.0) | 125/128 (97.7) | 91/93 (97.9) | 109/112 (97.3) | 122/124 (98.4) | 12/12 (100) |

Suppl. Table 3B: **Maximum 6-hour PEW**

| PEW score | Alder Hey | Bedside | Bristol | Irish | National | Newcastle | Scottish |
| --- | --- | --- | --- | --- | --- | --- | --- |
| 0 | 7/298 (2.3) | 3/93 (3.2) | 3/88 (3.4) | 4/108 (3.7) | 2/57 (3.5) | 3/104 (2.8) | 5/142 (3.5) |
| 1 | 14/115 (12.2) | 9/175 (5.1) | 6/124 (4.8) | 5/148 (3.4) | 7/132 (5.3) | 6/175 (3.4) | 11/152 (6.0) |
| 2 | 19/102 (18.6) | 12/135 (8.9) | 6/127 (4.7) | 4/94 (4.3) | 9/153 (5.9) | 7/107 (6.5) | 20/79 (25.3) |
| 3 | 16/31 (51.6) | 8/63 (12.7) | 7/55 (12.7) | 7/72 (9.7) | 5/61 (8.2) | 8/61 (13.1) | 14/70 (20.0) |
| 4 | 23/30 (76.7) | 13/45 (25.9) | 14/56 (25.0) | 8/50 (16.0) | 14/47 (29.8) | 8/34 (23.5) | 34/74 (45.9) |
| 5 | 33/33 (100) | 17/34 (50.0) | 7/42 (16.7) | 13/37 (35.1) | 15/51 (29.4) | 16/34 (47.1) | 16/31 (51.6) |
| 6 | 27/28 (96.4) | 28/38 (73.7) | 12/34 (35.3) | 18/31 (58.1) | 20/45 (44.4) | 17/30 (56.7) | 34/51 (66.7) |
| 7 | 29/31 (93.5) | 14/18 (77.8) | 19/30 (63.3) | 23/28 (82.1) | 19/27 (70.4) | 17/25 (68.0) | 28/40 (70.0) |
| 8 | 26/26 (100) | 19/20 (90.0) | 26/35 (74.3) | 31/41 (75.6) | 25/33 (75.8) | 14/16 (87.5) | 29/34 (85.3) |
| 9 | 24/24 (100) | 32/32 (100) | 16/22 (72.7) | 36/38 (94.7) | 19/26 (73.1) | 22/25 (88.0) | 37/39 (94.9) |
| 10+ | 30/30 (100) | 94/95 (99.0) | 124/127(97.6) | 98/100 (98.0) | 110/113 (97.4) | 110/114 (96.5) | 19/20 (95.0) |

Suppl. Table 3C: **Maximum 12-hour PEW**

| PEW score | Alder Hey | Bedside | Bristol | Irish | National | Newcastle | Scottish |
| --- | --- | --- | --- | --- | --- | --- | --- |
| 0 | 2/217 (0.9) | 0/51 | 0/43 | 0/60 | 0/27 | 1/62 (1.6) | 1/81 (1.2) |
| 1 | 12/152 (7.9) | 4/152 (2.6) | 2/94 (2.1) | 3/129 (2.3) | 2/98 (2.0) | 2/154 (1.3) | 5/151 (3.3) |
| 2 | 20/121 (16.5) | 10/148 (6.3) | 5/132 (3.8) | 5/110 (4.5) | 9/160 (5.5) | 8/135 (5.9) | 20/98 (20.4) |
| 3 | 16/44 (36.4) | 14/87 (16.1) | 20/55 (56.3) | 7/80 (8.8) | 8/74 (10.8) | 8/65 (12.3) | 15/92 (16.3) |
| 4 | 23/34 (67.6) | 8/54 (14.3) | 13/66 (19.7) | 9/62 (14.5) | 15/64 (23.4) | 9/43 (20.9) | 30/94 (31.9) |
| 5 | 33/34 (97.1) | 15/38 (47.4) | 6/45 (13.3) | 13/51 (25.5) | 11/50 (22.0) | 12/37 (32.4) | 37/64 (57.8) |
| 6 | 27/28 (96.4) | 27/41 (65.9) | 13/45 (28.9) | 14/34 (41.2) | 20/49 (40.8) | 14/37 (37.8) | 28/44 (63.5) |
| 7 | 31/33 (93.9) | 19/23 (82.6) | 18/32 (56.3) | 22/32 (68.9) | 19/36 (52.8) | 20/28 (71.4) | 25/33 (75.8) |
| 8 | 28/28 (100) | 15/17 (88.2) | 24/36 (66.7) | 32/41 (78.0) | 19/28 (67.9) | 13/16 (81.3) | 39/41 (95.1) |
| 9 | 23/23 (100) | 32/34 (94.1) | 16/24 (66.7) | 39/41 (95.1) | 26/34 (76.5) | 22/27 (81.5) | 23/24 (95.8) |
| 10+ | 31/32 (96.9) | 96/98 (98.0) | 143/148(96.6) | 104/108 (96.3) | 116/121 (95.9) | 120/125 (96.0) | 27/28 (96.4) |

Suppl. Table 3D: **Maximum 24-hour PEW**

| PEW score | Alder Hey | Bedside | Bristol | Irish | National | Newcastle | Scottish |
| --- | --- | --- | --- | --- | --- | --- | --- |
| 0 | 2/156 (1.3) | 0/33 | 0/29 | 0/37 | 0/15 | 1/33 (2.9) | 1/50 (2.0) |
| 1 | 9/168 (5.4) | 4/103 (3.4) | 1/62 (1.6) | 3/97 (3.1) | 2/62 (3.1) | 2/123 (1.6) | 4/120 (3.3) |
| 2 | 19/132 (14.4) | 9/158 (5.7) | 5/102 (4.9) | 3/98 (3.1) | 7/136 (5.1) | 6/127 (4.7) | 17/88 (19.3) |
| 3 | 14/58 (24.1) | 12/102 (11.8) | 8/95 (8.4) | 6/95 (6.3) | 7/91 (7.7) | 7/94 (8.3) | 9/106 (8.5) |
| 4 | 25/43 (58.1) | 9/65 (13.8) | 12/81 (14.8) | 11/77 (14.3) | 14/71 (19.7) | 10/61 (16.4) | 27/110 (24.5) |
| 5 | 31/35 (88.6) | 16/51 (31.4) | 9/59 (15.3) | 12/55 (21.8) | 11/66 (16.7) | 13/42 (31.0) | 35/73 (47.9) |
| 6 | 29/32 (90.6) | 26/43 (60.5) | 13/56 (23.2) | 14/41 (34.1) | 18/59 (30.5) | 9/43 (20.9) | 30/52 (57.1) |
| 7 | 31/33 (93.9) | 17/41 (70.8) | 17/37 (46.9) | 18/39 (46.2) | 20/39 (51.3) | 20/30 (66.7) | 25/38 (65.8) |
| 8 | 29/30 (96.7) | 12/17 (70.6) | 20/40 (50.0) | 31/44 (70.5) | 16/30 (53.3) | 16/22 (72.7) | 45/51 (88.2) |
| 9 | 26/27 (96.3) | 31/34 (91.2) | 15/25 (60.0) | 36/42 (85.7) | 24/59 (68.5) | 17/22 (77.3) | 27/30 (90.0) |
| 10+ | 35/36 (97.2) | 113/119 (95.0) | 150/164(91.5) | 116/125 (92.9) | 131/144 (91.0) | 149/162 (92.0) | 30/32 (93.8) |

**
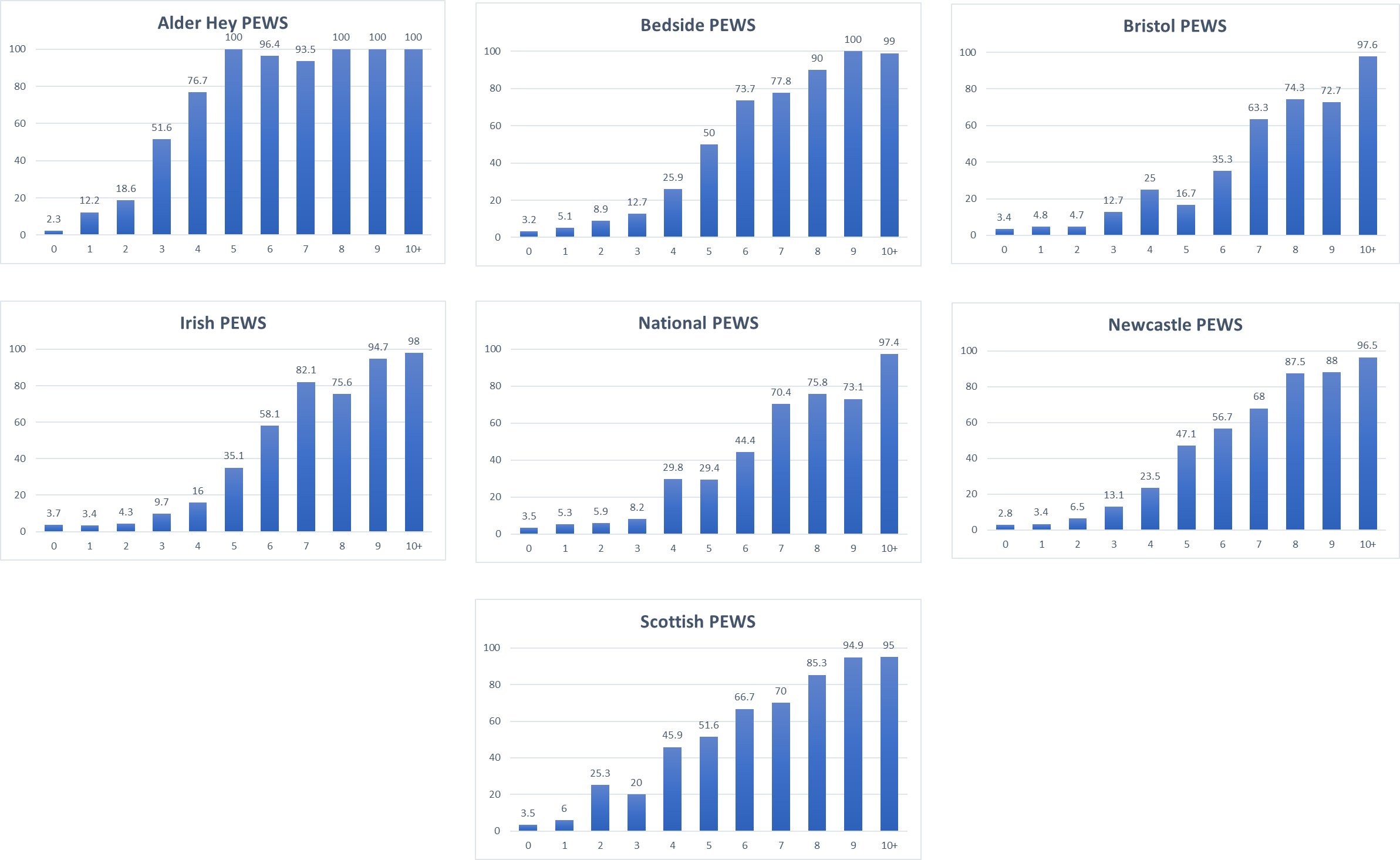
Suppl Figure 1: Frequency of CDE within 6 hours of worst PEWS a) Alder Hey PEWS, b) Bedside PEWS, c) Bristol PEWS, d) Irish PEWS, e) Proposed National PEWS for England, f) Newcastle PEWS, g) Scottish PEWS**

**Suppl. Figure 2 – Kaplain-Meir Survival Curves Demonstrating Time to CDE Using Sensitivity Thresholds at:**

**i: 4 Hours ii: 12 Hours**


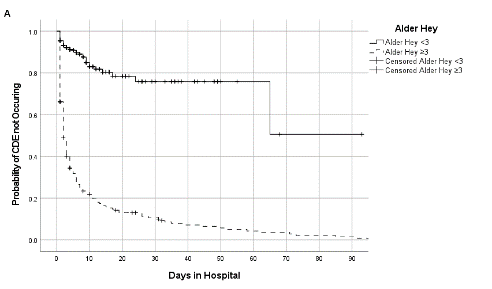

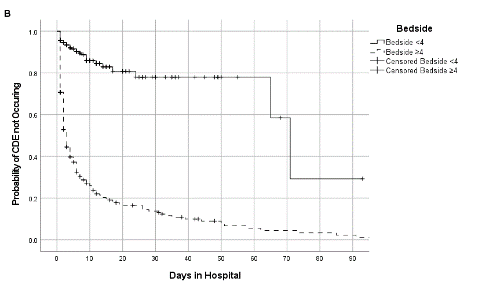

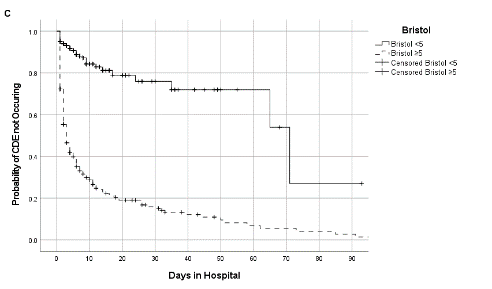

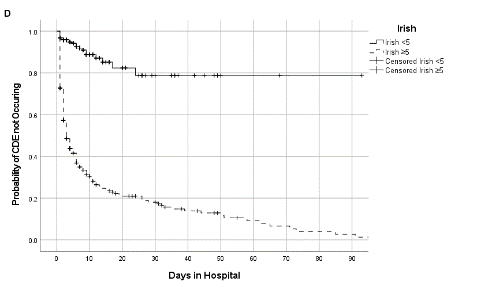

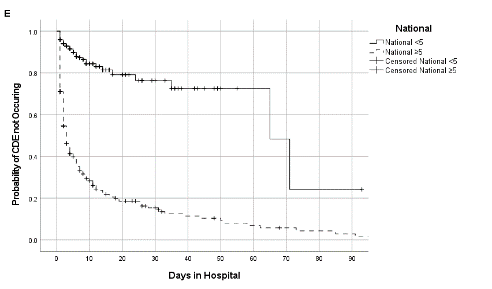

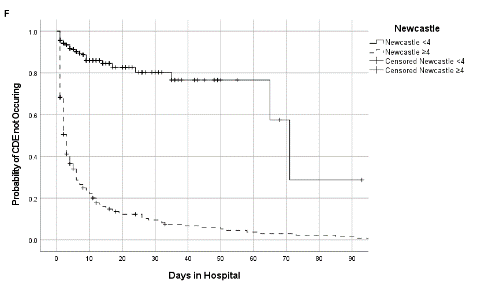

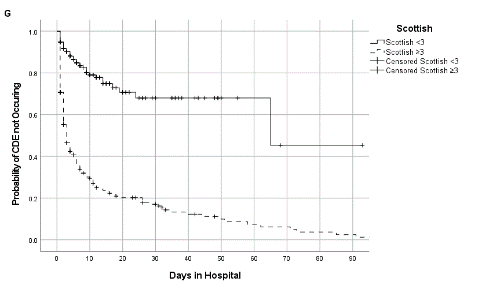

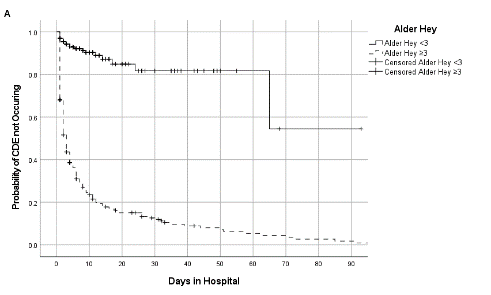

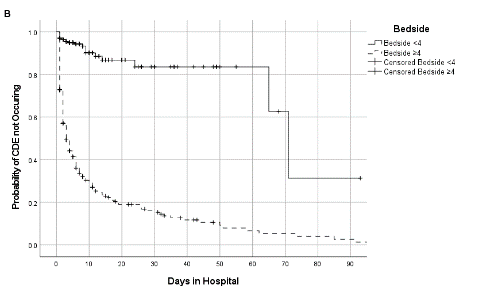

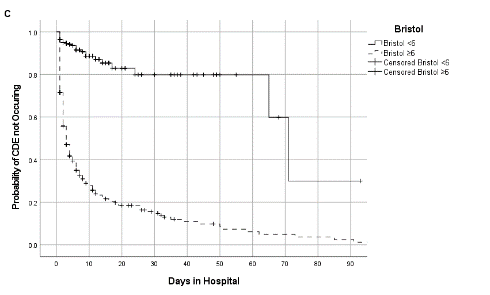

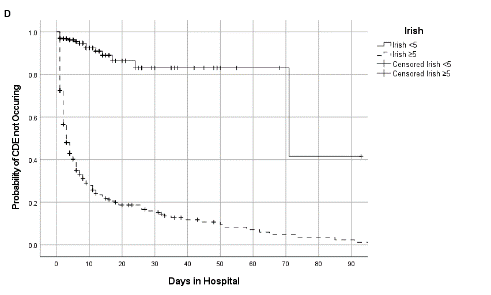

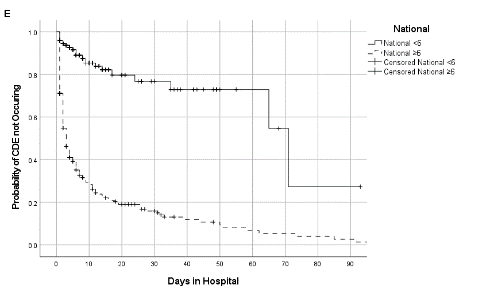

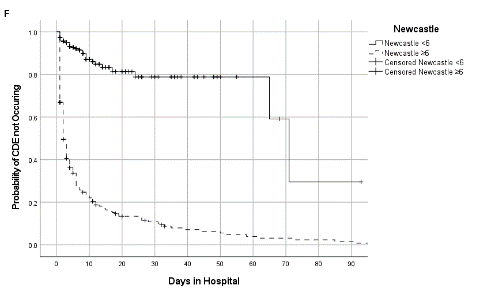

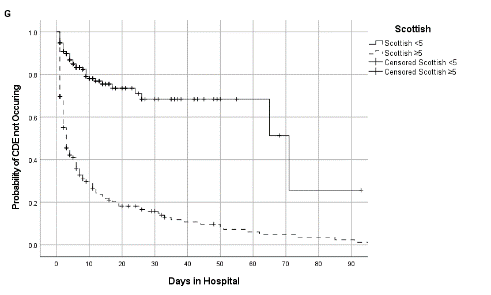


**iii: 24 Hours**


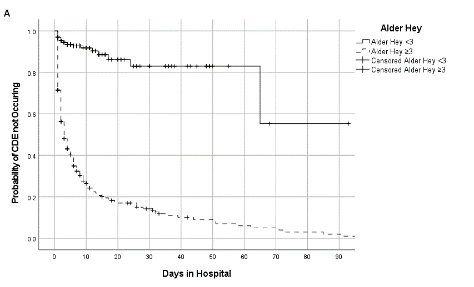

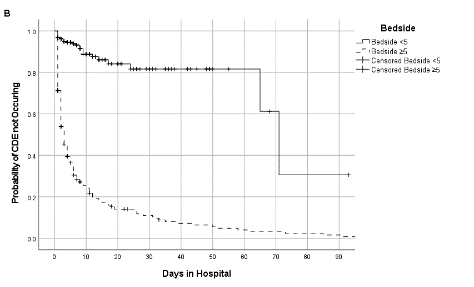

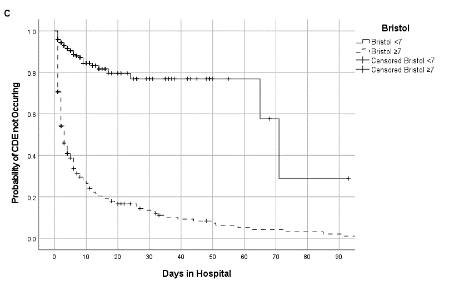

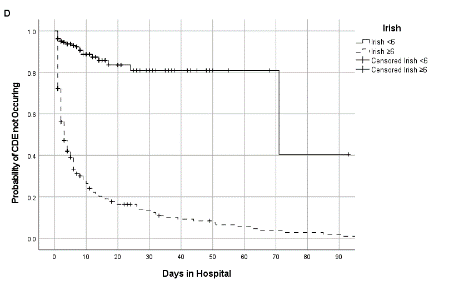

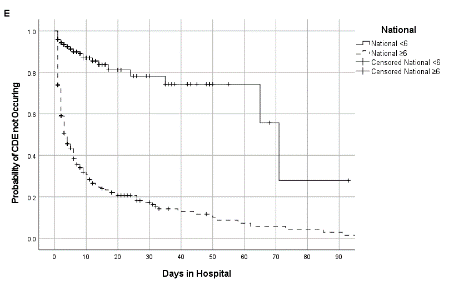

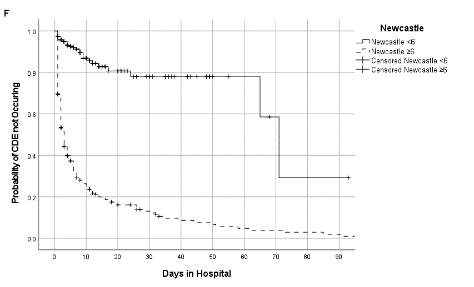

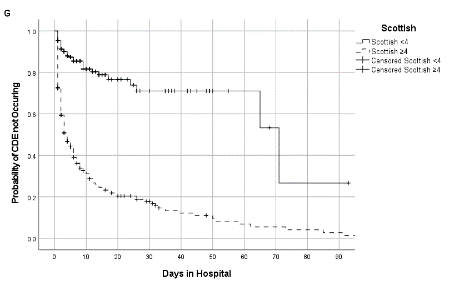


**Suppl. Table 4: Whole Population Characteristics**

| **Specialty** | **Number of Patient Visits** | **% of Total Population** | **Number of CDEs** | **% of total CDEs** | **% of total specialty** |
| --- | --- | --- | --- | --- | --- |
| General Paediatrics | 5569 | 31.79 | 89 | 35.6 | 1.6 |
| Haematology Oncology | 2682 | 15.31 | 14 | 5.6 | 0.52 |
| General Surgery | 2369 | 13.52 | 12 | 4.8 | 0.51 |
| Orthopaedics | 1419 | 8.1 | 3 | 1.2 | 0.21 |
| Cardiology | 1045 | 5.96 | 59 | 23.6 | 5.65 |
| ENT | 899 | 5.13 | 8 | 3.2 | 0.89 |
| Plastics & Burns | 846 | 4.83 | 1 | 0.4 | 0.12 |
| Neurosurgery | 598 | 3.41 | 3 | 1.2 | 0.5 |
| Respiratory | 578 | 3.3 | 38 | 15.2 | 6.57 |
| Maxillofacial | 448 | 2.56 | 0 | 0 | 0 |
| Neurology | 329 | 1.88 | 17 | 6.8 | 5.17 |
| Gastroenterology | 305 | 1.74 | 4 | 1.6 | 1.31 |
| Renal | 161 | 0.92 | 2 | 0.8 | 1.24 |
| Endocrine | 80 | 0.46 | 0 | 0 | 0 |
| Radiology | 73 | 0.42 | 0 | 0 | 0 |
| Rheumatology | 54 | 0.31 | 0 | 0 | 0 |
| Ophthalmology | 53 | 0.3 | 0 | 0 | 0 |
| Infectious Diseases | 11 | 0.06 | 0 | 0 | 0 |

**Suppl. Table 5: Performance of maximum PEWS to predict CDE in sub-specialty patients at:**

1. **4 Hours:**

| PEW | General Paediatrics | Cardiology | Respiratory |
| --- | --- | --- | --- |
|  | Area under ROC (95% confidence interval) | Area under ROC (95% confidence interval) | Area under ROC (95% confidence interval) |
| Alder Hey | 0.97 (0.94, 0.99) | 0.94 (0.90, 0.97) | 0.94 (0.89, 0.99) |
| Bedside | 0.96 (0.94, 0.99) | 0.87 (0.81, 0.93) | 0.91 (0.85, 0.97) |
| Bristol | 0.96 (0.94, 0.99) | 0.90 (0.85, 0.95) | 0.93 (0.87, 0.98) |
| Irish | 0.97 (0.95, 0.99) | 0.91 (0.86, 0.96) | 0.94 (0.89, 0.99) |
| National | 0.96 (0.93, 0.99) | 0.88 (0.82, 0.94) | 0.91 (0.84, 0.97) |
| Newcastle | 0.96 (0.94, 0.99) | 0.92 (0.88, 0.96) | 0.93 (0.87, 0.99) |
| Scottish | 0.90 (0.87, 0.94) | 0.88 (0.83, 0.93) | 0.86 (0.78, 0.93) |

1. **6 Hours:**

| PEW | General Paediatrics | Cardiology | Respiratory |
| --- | --- | --- | --- |
|  | Area under ROC (95% confidence interval) | Area under ROC (95% confidence interval) | Area under ROC (95% confidence interval) |
| Alder Hey | 0.98 (0.96, 0.99) | 0.93 (0.89, 0.97) | 0.94 (0.89, 0.99) |
| Bedside | 0.97 (0.94, 0.99) | 0.89 (0.83, 0.95) | 0.93 (0.88, 0.98) |
| Bristol | 0.97 (0.94, 0.99) | 0.87 (0.81, 0.93) | 0.91 (0.86, 0.97) |
| Irish | 0.97 (0.95, 0.99) | 0.90 (0.85, 0.95) | 0.94 (0.89, 0.99) |
| National | 0.96 (0.93, 0.98) | 0.82 (0.90, 0.94) | 0.91 (0.85, 0.97) |
| Newcastle | 0.98 (0.96, 0.99) | 0.91 (0.86, 0.96) | 0.93 (0.87, 0.99) |
| Scottish | 0.92 (0.88, 0.95) | 0.87 (0.82, 0.92) | 0.86 (0.79, 0.93) |

1. **12 Hours:**

| PEW | General Paediatrics | Cardiology | Respiratory |
| --- | --- | --- | --- |
|  | Area under ROC (95% confidence interval) | Area under ROC (95% confidence interval) | Area under ROC (95% confidence interval) |
| Alder Hey | 0.98 (0.96, 0.99) | 0.93 (0.89, 0.97) | 0.95 (0.91, 0.99) |
| Bedside | 0.97 (0.95, 0.99) | 0.89 (0.83, 0.95) | 0.95 (0.92, 0.99) |
| Bristol | 0.97 (0.95, 0.99) | 0.89 (0.81, 0.93) | 0.94 (0.90, 0.98) |
| Irish | 0.98 (0.96, 0.99) | 0.89 (0.84, 0.95) | 0.96 (0.92, 0.99) |
| National | 0.96 (0.93, 0.98) | 0.88 (0.82, 0.93) | 0.92 (0.86, 0.97) |
| Newcastle | 0.97 (0.95, 0.99) | 0.90 (0.85, 0.95) | 0.96 (0.92, 0.99) |
| Scottish | 0.91 (0.87, 0.95) | 0.85 (0.80, 0.91) | 0.87 (0.80, 0.93) |

1. **24 Hours:**

| PEW | General Paediatrics | Cardiology | Respiratory |
| --- | --- | --- | --- |
|  | Area under ROC (95% confidence interval) | Area under ROC (95% confidence interval) | Area under ROC (95% confidence interval) |
| Alder Hey | 0.96 (0.94, 0.99) | 0.91 (0.86, 0.95) | 0.95 (0.92, 0.99) |
| Bedside | 0.94 (0.91, 0.97) | 0.87 (0.80, 0.93) | 0.95 (0.92, 0.99) |
| Bristol | 0.94 (0.91, 0.97) | 0.84 (0.77, 0.90) | 0.94 (0.89, 0.98) |
| Irish | 0.95 (0.92, 0.98) | 0.86 (0.80, 0.92) | 0.96 (0.92, 0.99) |
| National | 0.94 (0.90, 0.97) | 0.85 (0.79, 0.91) | 0.91 (0.86, 0.97) |
| Newcastle | 0.95 (0.92, 0.97) | 0.89 (0.83, 0.94) | 0.96 (0.93, 0.99) |
| Scottish | 0.90 (0.86, 0.94) | 0.82 (0.75, 0.88) | 0.87 (0.80, 0.94) |
